# Laparoscopy With Percutaneous Transgastric Endoscopy for the Assessment of Gastric Cancer in the Excluded Stomach of a Roux-en-Y Gastric Bypass Patient

**DOI:** 10.7759/cureus.62727

**Published:** 2024-06-19

**Authors:** William A Baker, Danuel Laan

**Affiliations:** 1 Bariatric and General Surgery, USA Health Providence Hospital, Mobile, USA

**Keywords:** signet ring cell adenocarcinoma, percutaneous transgastric endoscopy, laparoscopy, excluded gastric remnant, la-ercp, roux-en y, case report, gastric cancer

## Abstract

Primary adenocarcinoma in the excluded stomach of Roux-en-Y gastric bypass (RYGB) patients is extremely rare. As such, the most effective diagnostic approach has not yet been determined. In typical patients, endoscopic ultrasound (EUS) is the first-line technique for evaluating suspected gastric cancer. However, RYGB patients require a more personalized approach. Endoscopic evaluation of the excluded stomach in RYGB patients, whether by EUS-directed, enteroscopy-assisted, or percutaneous means, is undoubtedly more complex than in patients with normal anatomy. In addition, gastric cancer is often diagnosed at an advanced stage due to its asymptomatic early course. With the added complexity of endoscopy in RYGB patients, a laparoscopic-assisted endoscopic approach may have a more favorable diagnostic and therapeutic utility in the case of gastric remnant malignancy in RYGB patients. The following case describes this comprehensive laparoscopic and endoscopic approach for the diagnosis of gastric cancer of the excluded stomach in an RYGB patient.

## Introduction

Primary adenocarcinoma in the excluded stomach of Roux-en-Y gastric bypass (RYGB) patients is extremely rare, with only a handful of reported cases in current medical literature. Due to the surgically altered anatomy of these patients, the most effective diagnostic procedure may differ from that of patients with normal anatomy.

Endoscopic ultrasound (EUS, echoendoscopy) is the preferred initial approach for diagnosing gastric cancer in patients with normal anatomy; however, endoscopy of an RYGB patient is inherently more complex. EUS-directed transgastric ERCP (EDGE) is quickly becoming the preferred technique for obtaining endoscopic access to the excluded stomach in RYGB patients, and derivative EUS-directed transgastric interventions (EDGIs) have even been used to diagnose gastric cancer in the excluded stomach [1-2]. Alternative techniques for accessing the excluded stomach in RYGB patients include double-balloon enteroscopy (DBE), laparoscopy-assisted (LA) transgastric endoscopy, and image-guided percutaneous techniques using ultrasound or fluoroscopy.​ These approaches are more often used for endoscopic retrograde cholangiopancreatography (ERCP) in RYGB patients with hepatopancreaticobiliary complaints; however, “EDGE” and “LA-ERCP” are often used interchangeably with “EUS-directed transgastric endoscopy” and “laparoscopic-assisted transgastric endoscopy,” respectively, in reference to the method of obtaining access to the excluded stomach rather than the specific endoscopic indication.

Of these techniques, EDGE and LA-ERCP have demonstrated comparably high efficacy, both with low rates of adverse outcomes [3]. While EDGE offers a less invasive means of endoscopy than LA-ERCP, it is undoubtedly more complicated than a routine EUS procedure in patients with normal anatomy, which can be performed in an outpatient setting under moderate or deep sedation [4]. EDGE often requires the patient to be placed under general anesthesia, and the endoscopist must deploy a lumen-apposing metal stent (LAMS) to create a fistulous tract between the gastric pouch or proximal jejunum and the excluded stomach. Given this added complexity of EUS in RYGB patients, the question arises as to whether the evaluation of gastric cancer of the excluded stomach should follow the same algorithm as anatomically normal patients. The answer to this question should be informed by the patient’s overall health status and suspected extent of disease at presentation combined with an adequate understanding of the indications and limitations of endoscopy in the diagnosis of gastric malignancy.

The following case describes the successful diagnosis and palliative intervention of metastatic gastric adenocarcinoma in the excluded stomach in an RYGB patient using a combined laparoscopic and endoscopic approach. The benefits of a combined procedure versus endoscopy alone will be discussed as it pertains to gastric cancer in the excluded stomach of an RYGB patient.

## Case presentation

A 79-year-old female was evaluated in the emergency department for sharp, burning lower abdominal pain that began two weeks earlier. She denied nausea or vomiting but reported a weight loss of 20 pounds in the last two months. Her medical history was notable for alcohol abuse disorder. Surgical history was significant for an open RYGB 20 years earlier, laparoscopic cholecystectomy, ventral hernia repair with mesh, and a panniculectomy.

She was admitted to the hospital for further workup. Liver function tests suggested posthepatic cholestasis and multiple gastrointestinal tumor markers, including carcinoembryogenic antigen (CEA), serum cancer antigen 19-9 (CA-19-9), and cancer antigen 125 (CA-125), were elevated. AFP levels were normal (Table 1).

**Table 1:** Liver function tests and serum tumor markers ALT: alanine transaminase, AST: aspartate transaminase, CEA: carcinoembryogenic antigen, CA-19-9: cancer antigen 19-9, CA-125: cancer antigen 125, AFP: alpha-fetoprotein

Serum marker	Value	Reference range
ALT	138 U/L	0-40 U/L
AST	141 U/L	0-40 U/L
Alkaline phosphatase	691 U/L	30-115 U/L
Bilirubin, total	2.0 mg/dL	0-1.2 mg/dL
CEA, nonsmoker	14 ng/L	0-3 ng/L
CA-19-9	479 U/mL	0-37 U/mL
CA-125	71 U/mL	0-35 U/mL
AFP	3.1 ng/mL	0-20 ng/L

**Figure 1:**
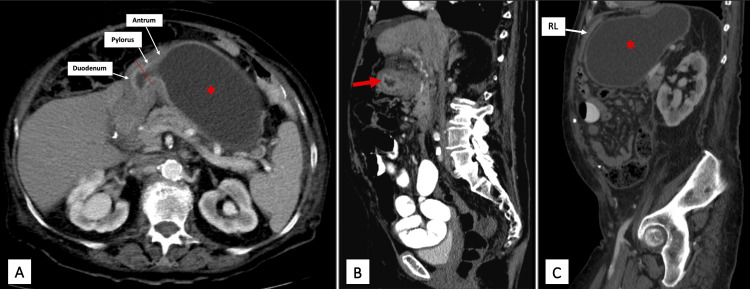
Abdominal CT scan with contrast 1A: Axial view of the distended fluid-filled excluded stomach (*) with contiguous thickening of the antrum, pylorus, and proximal duodenum (arrows). The stenotic pyloric sphincter is bisected by the dashed red line. 1B: Sagittal view showing mucosal thickening at the proximal duodenum (red arrow). 1C: Impingement of the Roux limb (RL) by the distended excluded stomach (*).

An abdominal CT scan (Figure 1) revealed retroperitoneal lymphadenopathy and a dilated fluid-filled gastric remnant with a markedly thickened antrum, pylorus, and proximal duodenum. Magnetic resonance cholangiopancreatography (MRCP) showed no evidence of bile duct obstruction or dilatation despite elevation in aspartate transaminase (AST), alanine transaminase (ALT), alkaline phosphatase, and bilirubin. With these findings, a surgical consult was made, and diagnostic laparoscopy with percutaneous transgastric endoscopy was performed.

Abdominal exploration

**Figure 2:**
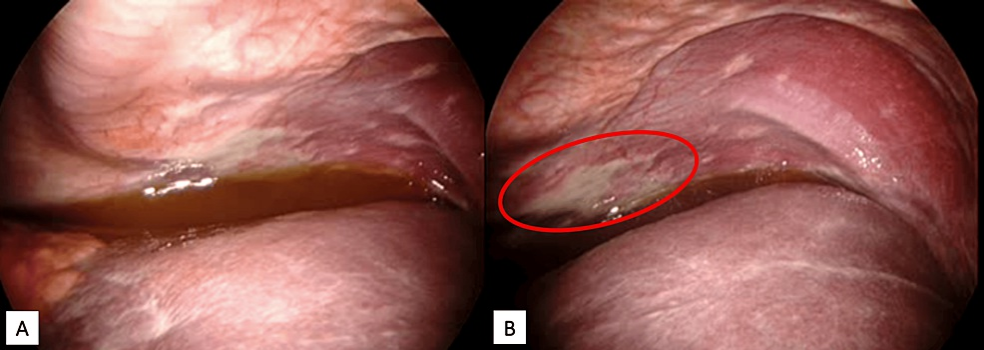
Laparoscopic exploration of the peritoneal cavity 2A: Dark yellow ascitic fluid pooling dependently in the abdomen. 2B: Patchy white lesions on anterior abdominal wall, later confirmed as peritoneal metastases.

Upon initial inspection of the peritoneal cavity, dark-yellow ascitic fluid was observed within the abdomen (Figure 2A). There were also a number of abnormal-appearing white deposits on the anterior peritoneal wall (Figure 2B). Samples were collected and sent for pathologic examination.

**Figure 3:**
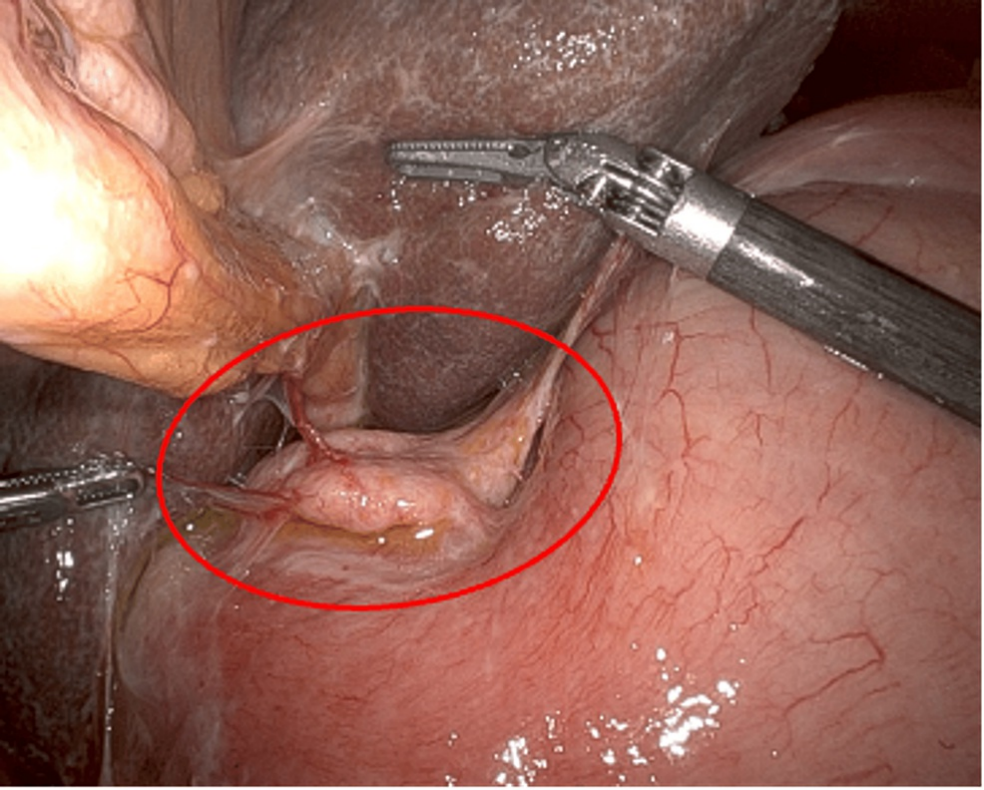
Lesser curvature of the excluded stomach Vascularized exophytic mass along the lesser curvature of the excluded stomach.

The entire length of the roux limb was healthy in appearance. Peterson’s space was identified, and there was no indication of mesenteric defects or internal herniation. The liver was healthy in appearance with a surgically absent gallbladder. The excluded stomach was grossly distended with a vascularized firm, rubbery mass along the lesser curvature (Figure 3). A sample was biopsied with scissors and sent for pathology.

Gastrostomy for endoscopic access to the excluded stomach

**Figure 4:**
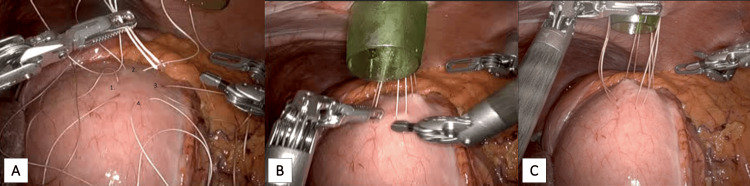
Securing the remnant stomach to the laparoscopic access port 4A: Four stay sutures placed in a diamond pattern on the superolateral greater curvature of the remnant stomach. 4B: Free ends of the sutures were pulled through the laparoscopic incision as the 15 mm trochar was advanced. 4C: Sutures were arranged evenly around the trochar and loosely tensioned.

The excluded stomach was approximated to the anterior abdominal wall (Figure 4) by four circumferential stay sutures, leaving room for a central gastrotomy incision. A 15 mm trochar was placed in the left upper quadrant of the abdomen in proximity to the excluded stomach, and the stay sutures were pulled through the laparoscopic incision along the outside of the trochar.

**Figure 5:**
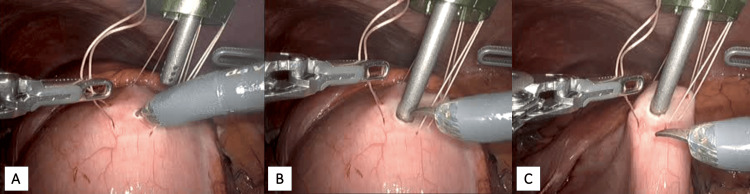
Drainage of the remnant stomach 5A: A very small gastrotomy incision was made, just large enough to accommodate the suction irrigation tip. 5B: Suction tip was advanced 1-2 cm into the fluid-filled remnant stomach. 5C: The stomach was completely drained of fluid, which was similar in appearance to the ascitic fluid seen in the abdomen.

Next, a small gastrotomy incision was made. A laparoscopic suction irrigation tip was carefully advanced, and the remnant stomach was completely drained (Figure 5). Fluid was sent for pathological analysis.

**Figure 6:**
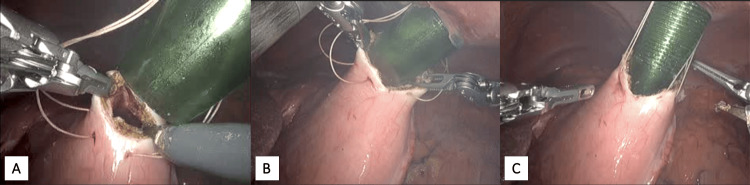
Gastrostomy for endoscopic access 6A: Widening of gastrotomy to accommodate the 15 mm trochar. 6B: Advancing the 15 mm trochar into the excluded stomach. 6C: Stay sutures are tensioned and fastened.

The gastrotomy incision was widened to accommodate the 15 mm trochar, which was advanced into the excluded stomach (Figure 6). The four stay sutures were tensioned and secured outside of the abdomen.

Percutaneous transgastric endoscopy

**Figure 7:**
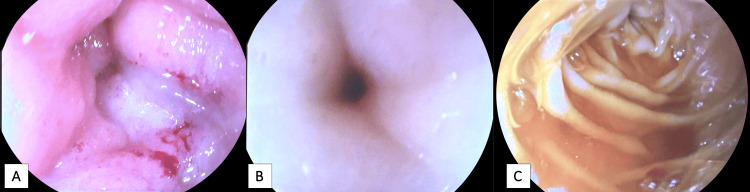
Endoscopy of the excluded stomach and duodenum 7A: View of the gastric antrum. 7B: View of the pyloric sphincter. 7C: Complete obstruction at the distal duodenum.

Additional sterile drapes were placed to exclude the 15 mm trochar from the sterile surgical field and endoscopy was performed. The gastric mucosa had an abnormal appearance throughout. There was diffuse erythema and a polypoid lesion in the antrum, which was biopsied and sent for pathology. Continuing distally, the antrum became increasingly narrowed. The pylorus was significantly thickened and stenotic (Figure 7A, 7B). With gentle pressure, we were able to advance beyond the pylorus to observe a normal-appearing proximal duodenum and ampulla of Vater; however, there was an additional narrowing in the distal duodenum causing a complete obstruction (Figure 7C). and we were unable to advance the endoscope any further. The regular endoscope was removed and replaced by an echoendoscope, which revealed significant lymphadenopathy in the region of the porta hepatis; however. needle biopsies were not obtained due to difficulty maintaining the position of the endoscope in this location.

Gastrotomy closure with tangential partial gastrectomy

The endoscope and 15 mm trochar were withdrawn from the excluded stomach. A partial gastrectomy was performed just proximal to the portion of the fundus where endoscopic access was obtained. SureForm 60 mm black load robotic stapler (Intuitive Surgical, Inc., USA) with staple line reinforcement was used to seal the remnant stomach and the resected portion was sent for pathology. 

Pathology and postoperative course

Histopathological analysis was conducted concurrently as biopsy samples were obtained. Nearing completion of endoscopic evaluation of the excluded stomach, results of initial peritoneal tissue and ascitic fluid biopsies confirmed signet ring cells with positive staining to cytokeratin, cytokeratin 7, and cytokeratin 20 in all samples. At this time, the endoscope was withdrawn, a palliative gastrostomy was placed, and surgery was concluded. Gastric mucosal biopsies later confirmed signet ring cells, and the patient was diagnosed with metastatic gastric adenocarcinoma. Over the next six days, she recovered well in the hospital without any complications; however, given her advanced disease, the patient died two months later in hospice care.

## Discussion

Early-stage, gastric cancer is largely asymptomatic, and most patients have advanced-stage disease at initial diagnosis [5]. Routine surveillance endoscopy of non-malignant lesions is not standard practice in countries with a low incidence of gastric cancers, such as the United States [6]. Therefore, patients often seek medical treatment only after the emergence of secondary alarm symptoms, such as dysphagia or weight loss [7]. By this time, their disease has likely evolved beyond that of an endoscopically resectable tumor.

Similarly insidious are the elevations in serum tumor markers in gastric cancer. Early-stage, resectable gastric tumors do not typically display elevated tumor markers [8], while elevations in CEA, CA19-9, and CA72-4 have been associated with more advanced disease and poorer prognosis in gastric cancers [8-10]. In a 2014 systematic review by the Task Force of the Japanese Gastric Cancer Association, Shimada et al. found elevated CA19-9 to have a positive predictive value of 78-96% for the presence of lymph node metastasis, while elevated CA-125 and AFP were noted to be associated with peritoneal and liver metastasis, respectively [11].

In patients with normal anatomy, endoscopic ultrasound (EUS) is useful for diagnosing, staging, and resecting early gastric lesions. The depth of the primary lesion can be assessed accurately by echoendoscope, and partial thickness (mucosal) tumors can be resected endoscopically [12-14]. EUS can also determine local lymph node involvement; however, compared to laparoscopy, N-staging of primary gastric cancers by EUS is often underestimated [15]. Once the tumor invades beyond the mucosa, the diagnostic and therapeutic yield of EUS (and therefore EDGE) declines precipitously [16-18]. For locally advanced or large primary tumors, gastrectomy with D2 lymphadenectomy is the only potentially curative option [19].

Given that the majority of patients with gastric cancer in Western countries present with advanced-stage disease at initial diagnosis, a solely endoscopic technique, such as EDGE or double balloon enteroscopy, may be insufficient in determining the full extent of disease in RYGB patients and may require the patient to undergo additional diagnostic, staging, or palliative procedures at a later time. A laparoscopic approach not only allows for exploration of the abdomen for signs of lymph node, liver, and peritoneal metastases but also provides an option for intraoperative palliative interventions, such as gastrostomy placement or gastrectomy, if indicated.

In the case of our patient presenting with a 20-pound weight loss, elevated serum CEA, CA125, CA19-9, and a distended fluid-filled gastric remnant and retroperitoneal lymphadenopathy on preoperative CT scan, there was a high preoperative suspicion of advanced-stage gastric cancer. By performing a diagnostic laparoscopy with intraoperative transgastric endoscopy of the gastric remnant, we were able to obtain a diagnosis, confirm peritoneal metastasis, and provide relief for the patient's malignant gastric outlet obstruction, which would not have been possible by solely endoscopic means.

## Conclusions

Compared to endoscopic techniques, such as EDGE or double-balloon enteroscopy (DBE), laparoscopy with percutaneous transgastric endoscopy offers greater diagnostic and therapeutic utility for symptomatic RYGB patients in which a primary malignancy of the excluded stomach is suspected. The presence of alarm symptoms (dysphagia and weight loss), imaging evidence of malignant gastric outlet obstruction or lymph node involvement, and/or elevations in multiple serum tumor markers (CEA, CA19-9, and CA72-4) suggest more advanced disease. Thus, a laparoscopic approach ensures adequate assessment of the abdomen for lymph nodes, liver, and peritoneal metastases and offers the potential for curative resection or palliative intervention if indicated.

## References

[1] Kumar R, Pitea TC (2017). A novel endoscopic technique to diagnose gastric cancer in excluded stomach after Roux-en-Y Gastric bypass. ACG Case Rep J.

[2] Schneider L, Kröger A, Gubler C, The FO (2022). Diagnosis of gastric cancer in the excluded stomach after RYGB by jejunogastrostomy using a LAMS. ACG Case Rep J.

[3] Kedia P, Tarnasky PR, Nieto J (2019). EUS-directed transgastric ERCP (EDGE) versus laparoscopy-assisted ERCP (LA-ERCP) for Roux-en-Y gastric bypass (RYGB) anatomy: a multicenter early comparative experience of clinical outcomes. J Clin Gastroenterol.

[4] Dossa F, Megetto O, Yakubu M, Zhang DD, Baxter NN (2021). Sedation practices for routine gastrointestinal endoscopy: a systematic review of recommendations. BMC Gastroenterol.

[5] Schmidt N, Peitz U, Lippert H, Malfertheiner P (2005). Missing gastric cancer in dyspepsia. Aliment Pharmacol Ther.

[6] Smyth EC, Nilsson M, Grabsch HI, van Grieken NC, Lordick F (2020). Gastric cancer. Lancet.

[7] Kapoor N, Bassi A, Sturgess R, Bodger K (2005). Predictive value of alarm features in a rapid access upper gastrointestinal cancer service. Gut.

[8] Dilege E, Mihmanli M, Demir U (2010). Prognostic value of preoperative CEA and CA 19-9 levels in resectable gastric cancer. Hepatogastroenterology.

[9] Sun Z, Zhang N (2014). Clinical evaluation of CEA, CA19-9, CA72-4 and CA125 in gastric cancer patients with neoadjuvant chemotherapy. World J Surg Oncol.

[10] Feng F, Tian Y, Xu G (2017). Diagnostic and prognostic value of CEA, CA19-9, AFP and CA125 for early gastric cancer. BMC Cancer.

[11] Shimada H, Noie T, Ohashi M, Oba K, Takahashi Y (2014). Clinical significance of serum tumor markers for gastric cancer: a systematic review of literature by the Task Force of the Japanese Gastric Cancer Association. Gastric Cancer.

[12] Nunobe S, Kumagai K, Ida S, Ohashi M, Hiki N (2016). Minimally invasive surgery for stomach cancer. Jpn J Clin Oncol.

[13] Kuroki K, Oka S, Tanaka S (2021). Clinical significance of endoscopic ultrasonography in diagnosing invasion depth of early gastric cancer prior to endoscopic submucosal dissection. Gastric Cancer.

[14] Tsujii Y, Kato M, Inoue T (2015). Integrated diagnostic strategy for the invasion depth of early gastric cancer by conventional endoscopy and EUS. Gastrointest Endosc.

[15] Chen J, Zhou C, He M, Zhen Z, Wang J, Hu X (2019). A meta-analysis and systematic review of accuracy of endoscopic ultrasound for N staging of gastric cancers. Cancer Manag Res.

[16] Mocellin S, Pasquali S (2015). Diagnostic accuracy of endoscopic ultrasonography (EUS) for the preoperative locoregional staging of primary gastric cancer. Cochrane Database Syst Rev.

[17] Kim JH, Song KS, Youn YH, Lee YC, Cheon JH, Song SY, Chung JB (2007). Clinicopathologic factors influence accurate endosonographic assessment for early gastric cancer. Gastrointest Endosc.

[18] Shi D, Xi XX (2019). Factors affecting the accuracy of endoscopic ultrasonography in the diagnosis of early gastric cancer invasion depth: a meta-analysis. Gastroenterol Res Pract.

[19] (2021). Japanese gastric cancer treatment guidelines 2018 (5th edition). Gastric Cancer.

